# Faecal incontinence intervention study (FINS): self-management booklet information with or without nurse support to improve continence in people with inflammatory bowel disease: study protocol for a randomized controlled trial

**DOI:** 10.1186/s13063-015-0962-0

**Published:** 2015-10-06

**Authors:** Christine Norton, Lesley B. Dibley, Ailsa Hart, Julie Duncan, Anton Emmanuel, Charles H. Knowles, Natasha Stevens, Helen Terry, Azmina Verjee, Sally Kerry, Natalia Hounsome

**Affiliations:** Florence Nightingale Faculty of Nursing and Midwifery, King’s College, 57 Waterloo Road, London, SE1 8WA UK; St Mark’s Hospital, Watford Road, Harrow, HA1 3UJ UK; Guy’s and St Thomas’s NHS Trust, Westminster Bridge Road, London, SE1 7EH UK; University College Hospital, 235 Euston Road, London, NW1 2BU UK; Blizard Institute, Queen Mary, University of London, 4 Newark Street, London, E1 2AT UK; Crohn’s and Colitis UK, 4 Beaumont House, Sutton Road, St Albans, Hertfordshire AL1 5HH UK; Bowel Disease Research Foundation, The Royal College of Surgeons of England, 35-43 Lincoln’s Inn Fields, London, WC2A 3PE UK; Pragmatic Clinical Trials Unit, Blizard Institute, Queen Mary, University of London, 4 Newark Street, London, E1 2AT UK; Barts Health NHS Trust, 80 Newark Street, London, E1 2ES UK

**Keywords:** Inflammatory bowel disease, Faecal incontinence, Nurse-led intervention

## Abstract

**Background:**

Inflammatory bowel disease, comprising Crohn’s disease and ulcerative colitis, is a life-long currently incurable illness. It causes bouts of acute intestinal inflammation, in an unpredictable relapsing-remitting course, with bloody diarrhoea and extreme urgency to access a toilet. Faecal incontinence is a devastating social and hygiene problem, impacting heavily on quality of life and ability to work and socialise. Faecal incontinence affects 2–10 % of adults in the general population. People with inflammatory bowel disease have a high risk of incontinence with up to 74 % affected. No previous study has explored conservative interventions for these patients.

**Methods:**

This randomised controlled trial will recruit 186 participants to answer the research question: does implementation of the UK nationally recommended guidance approach to stepwise management of faecal incontinence improve bowel control and quality of life in people with inflammatory bowel disease? We have worked with people with inflammatory bowel disease to translate this guidance into a condition-specific information booklet on managing incontinence. We will randomise participants to receive the booklet, or the booklet plus up to four 30-minute sessions with an inflammatory bowel disease specialist nurse. To be eligible, patients must be in disease remission and report incontinence. The primary outcome measure at 6 months after randomisation is the St Mark’s incontinence score. Other outcomes include quality of life, MY-MOP (generic tool: participants set two goals for intervention, grading goals at baseline and then re-scoring after intervention) and EQ-5D-5 L to enable calculation of quality-adjusted life years. Analysis will be on an intention-to-treat basis. Qualitative interviews will explore participant and health professionals’ views on the interventions.

**Discussion:**

Few high-quality studies of conservative interventions in inflammatory bowel disease, and none for faecal incontinence, have been conducted. We have collaborated with patients to design this study. Blinding to this behavioural intervention is not possible, but our self-report outcome measures with a degree of objectivity. There is genuine equipoise between the booklet only and booklet plus nurse arms, and the study will determine if additional support from a nurse is a crucial element in implementing advice.

**Trial registration:**

clinitrials.gov.uk: NCT02355834 (Date of registration: 12 December 2014).

Protocol version: 4.0. 08.04.15

**Electronic supplementary material:**

The online version of this article (doi:10.1186/s13063-015-0962-0) contains supplementary material, which is available to authorized users.

## Background

### Background and rationale

Inflammatory bowel disease (IBD, comprising mostly Crohn’s disease and ulcerative colitis) is a life-long currently incurable disease affecting approximately 250,000 adults in the UK, 2.2 million in Europe and 1.4 million in North America [1]. It causes bouts of acute intestinal inflammation in an unpredictable relapsing-remitting pattern, with bloody diarrhoea and extreme urgency to access a toilet. Chronic diarrhoea, rectal inflammation and reduced rectal capacity, disease or surgery which affect the continence mechanism, and a fatigable anal sphincter [2] all contribute to a high risk of faecal incontinence (FI) [3].

FI is a devastating social and hygiene problem, impacting heavily on quality of life and ability to work and socialise, and affecting 2–10 % of adults in the general population [4, 5]. Urgency and FI are accepted by both patients and clinicians in IBD flare-up, but at least a quarter of people with IBD also report extremely limiting symptoms persisting in remission [3]. Some young adults are completely housebound [6]. Interviews with people with IBD-related FI (IBD-FI) describe multiple life limitations: ‘*the fear of being faecally incontinent has a great impact on your life, and… this fear turns into stress and anxiety, which make it more likely that you will be incontinent… you enter a vicious cycle. It makes you nervous about going out, using public transport, going on holiday, and having intimate relationships*’ [6].

The imperative to find a toilet immediately is as limiting as frank incontinence and is reported as happening daily by 89 % of people with IBD during a disease flare and 66 % in remission [7]. Poor bowel control and the need for urgent toilet access are among the top ten concerns of people with IBD [8]. Some 81.3 % of people with IBD in the UK worry about the availability of toilets whenever they go somewhere new [7]. Crohn’s and Colitis UK (patient charity) have prioritised the issue of FI in IBD, funding studies on prevalence and quality of life [3], experiences of IBD-FI [6], help-seeking [9], and development of a patient-reported questionnaire for IBD-FI [10].

Previous work on FI in non-IBD populations has found that 70–80 % cure and improvement is feasible. Patients receiving conservative management in specialist nurse services are highly satisfied with their improvement and report improved ability to live with symptoms and better quality of life [11–13]. IBD nurse specialists are widespread in the UK National Health Service (NHS) [14], are highly valued by patients, and view FI as one of their core areas of practice [15]. However, our qualitative and quantitative work [3, 6, 9] has found that few IBD nurses offer any intervention specifically for FI. Reasons for this are unclear, but informal discussions suggest the primary barrier is lack of training for IBD nurses in managing FI. Small studies have suggested some benefit for individual specialist interventions for IBD-FI but no comprehensive approach has been evaluated.

The UK National Institute for Health and Clinical Excellence (NICE) [13] recommends a step-wise intervention algorithm for FI, starting with low-cost conservative interventions such as diet and anti-diarrhoeal medication, bowel retraining and pelvic floor exercises, estimating that 70 % of people with FI can be improved. There are several Cochrane reviews finding that treatments are effective for FI [12, 16, 17]. None has included people with IBD. Co-author AE has found that 18/27 (66 %) IBD patients managed in a nurse-led FI service reported improved continence and 63 % a significantly reduced continence score (unpublished audit). In 218 patients with radiation bowel disease (with similar symptoms) it was found that nurse intervention improves FI and quality of life and produces similar response to gastroenterologist intervention and is significantly better than a booklet alone, with improvements sustained at 1 year [18]. Our specialist clinical experience suggests that people with IBD are likely to respond well to the NICE algorithmic approach.

### Objectives

We aim to answer the research question “Does implementation of the UK nationally (NICE)-recommended approach [13] to stepwise management of FI improve bowel control and quality of life in people with IBD?” Our objectives are to determine the effectiveness of using IBD nurses to deliver the algorithm of care proposed by NICE for people with FI, compared to provision of the same information in a self-management booklet and to obtain detailed qualitative feedback from patients and staff on the best way of enabling health-seeking, the experience of the intervention and suggestions for future service development.

### Trial design

This is a two-arm parallel group randomised controlled trial (RCT) with 1:1 allocation intended to show superiority of IBD nurse intervention plus an information booklet, compared with an information booklet alone. The study has a total duration of 30 months.

## Methods

### Study setting

Six hospital centres with IBD clinics in metropolitan, urban and rural locations in England are recruiting and treating participants, in partnership with a Pragmatic Clinical Trials Unit (PCTU) in London. A list of sites can be obtained from the lead author.

### Eligibility criteria

The *Screening Case Report Form* (*CRF*) will be used to conduct pre-registration evaluation of participants for eligibility.

#### Inclusion criteria

- Proof of IBD diagnosis (record of diagnostic endoscopy in patient notes).
- Between 18 and 80 years old
- No current flare-up of disease - stable disease and treatment status (in remission) for the past 3 months confirmed by documented normal results for inflammatory markers (C-reactive protein or faecal calprotectin [FCP]) recorded within a 3-month eligibility period; FCP tests to be repeated at recruitment to confirm IBD is in remission if no recent results documented
- Reporting FI at least once in the past year which is limiting self-reported quality of life and activities (determined by responses to survey above), enabling inclusion of the majority of people with IBD-FI whose quality of life is, in their own view, restricted

#### Exclusion criteria

- Current disease flare-up
- Course of specialist FI treatment in the past year (e.g. biofeedback, tibial nerve stimulation). From our previous work we hypothesise that this will be very few potential participants [4]
- Previous major anal fistula surgery or current perianal fistula (which can compromise anal sphincter function and control, increasing the risk of FI)
- Current stoma (faeces will be diverted)
- Current participation in another trial
- Inability to give informed consent (for example, due to reduced mental capacity)
- Inability to read or speak sufficient English to understand study documents, procedures and requirements.

### Interventions

***Group 1*(*IBD nurse CONSULT + BOOKLET*)**: will have 3–4 x 30-minute face-to-face sessions over 3 months with an IBD specialist nurse specifically focusing on bowel control. Participants completing at least three sessions will be considered to have completed the intervention. They will also be given the self-management booklet and access to all usual IBD care, including the routine local nurse-led IBD helpline.

#### Booklet development

We have worked with four people with IBD-FI to operationalise the UK NICE guidance on FI [13, 19] as a booklet for self-help for FI in IBD: *Managing Incontinence in Inflammatory Bowel Disease*. It includes practical advice on bowel habit, diet and fluids, anti-diarrhoeal medication, bowel retraining, pelvic floor exercises, practical coping and is based on our published advice for people with FI [20, 21] adapted to be IBD-specific.

#### Training IBD nurses

One or two IBD nurse specialists at each centre (maximum of 12 in total) will deliver the IBD nurse intervention. An intensive 1-day scripted training programme on managing FI will be delivered by two members of the study team. A checklist for intervention sessions included in the *Group 1 Intervention CRF* will monitor fidelity to the content and we will conduct two initial “test” sessions with each IBD nurse to gauge competence and 3–4 follow-up visits at random during patient consultations to gauge fidelity to the intervention.

***Group 2*(*BOOKLET alone*)**: will receive the same booklet and access to usual IBD care as group 1. We recognise the potential influence on usual care of training the IBD nurses to deliver the intervention. We will emphasise during training the importance of trying not to influence usual care for the booklet arm participants and wherever feasible for another team member to see these patients in consultations (teams are large enough to accommodate this). In reality the IBD nurses are very busy managing acute aspects of IBD and are unlikely to have time to spontaneously provide more intensive input for FI. Our interviews (below) will explore perceptions of whether usual care was influenced by having the trial in progress.

### Withdrawal criteria

Participants have the right to withdraw from the study at any time, despite having given consent to take part, without having to give a reason for doing so. Their right and access to their usual NHS treatment will not be compromised in any way if they do withdraw. As indicated on the *Patient Consent Form* and explained in the *Patient Information Sheet*, all data collected before the point of withdrawal will be retained and analysed unless the patient specifically requests otherwise. We will ask those who withdraw if their withdrawal is due to the conditions of the study (i.e. too onerous/difficult to adhere to), seeking a simple yes/no answer. No further detail will be sought. This information will help to place any attrition in context and inform ongoing intervention design beyond this study.

### Strategies to improve adherence

No specific strategies will be employed. We will explore perceived adherence and ease of complying with the advice in the nested qualitative study (below).

### Relevant concomitant care

Participants will have full access to usual NHS care for IBD during the trial.

### Study outcome measures

Data will be collected by a self-completed booklet of questionnaires, given to the participant by a research nurse not conducting the intervention. Study outcome measures will be recorded at baseline and at 6 months after randomisation.

#### Primary outcome measure

• The St Mark’s faecal incontinence score [22] (0–24 scale) at 6 months after randomisation, compared between the study groups, analysed as a continuous variable. This is a widely used outcome measure in studies of FI and a 3-point difference in score has been found to be clinically relevant [23].

#### Secondary outcome measures

- ICIQ–IBD (condition-specific questionnaire for symptoms and quality of life in IBD-FI) [10]
- Inflammatory bowel disease questionnaire [24] (the most widely used condition-specific IBD quality-of-life measure)
- Disease activity index to determine if disease activity level has changed (Harvey Bradshaw index for Crohn’s disease [25] and simple clinical colitis activity index for ulcerative colitis [26], and medical record of any escalation of treatment)
- MY-MOP [27] (generic tool: participants set two goals for intervention, grading goals at baseline and then re-scoring after intervention, giving an individualised profile of the most bothersome symptom, allowing analysis as a continuous variable)
- EQ-5D-5 L [28] to enable calculation of quality-adjusted life years (QALYS)
- Patient and NHS resource use for interventions (time and consumables used, time off work, travel costs)
- Study-specific tool: illness perceptions about FI and its treatability
- Brief illness perception score [29] to determine if illness perception influences completion and response to intervention
- Study-specific tool: global perception of change, which interventions participants engaged in (e.g. diet change, pelvic floor exercises, use of anti-diarrhoeal medication) and ease of compliance with advice, satisfaction with intervention and qualitative comments, any IBD flare-up (definition: need to escalate or initiate medication/dose) during or since intervention. This will enable insights into which elements of the intervention are perceived by patients as most important.

A copy of trial outcome measures is available from the lead author on request.

### Participant timeline

**Fig. 1:**
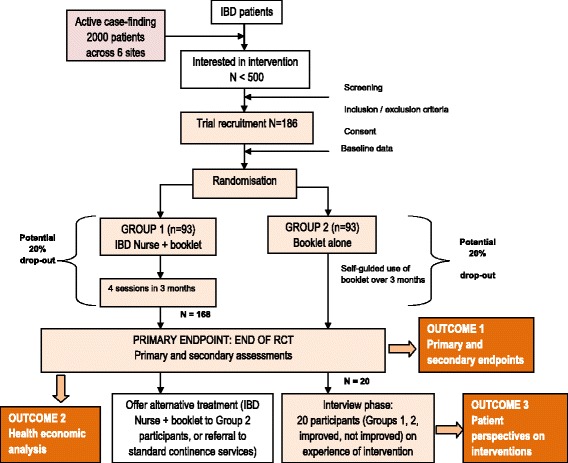
Patient flow diagram

The study flow chart is shown in Fig. 1.

### Sample size for the trial

Sample size is based on a published response (improvement) rate for conservative intervention for FI and a difference of 3 points in the St Mark’s FI score at 6 months being considered to be of clinical significance to people with FI but without IBD [23]. We will aim to randomise 186 people, with anticipated drop-out of 20 % (based on our previous intervention studies and clinical experience), which will be sufficient to detect a difference of 3 points on the St Mark’s scale assuming a standard deviation of 5, with a power of 90 %, using a 5 % significance level (74 needed per arm, increased to 93 to allow for drop-outs).

### Recruitment

We will conduct an initial active case-finding phase comprising a survey of the prevalence of FI among patients attending routine IBD clinics at the six centres. We aim to include at least 2,000 people in this survey. Respondents to this survey, who indicate a willingness to participate in the trial, will be contacted by the research nurse. Potential participants will be screened for eligibility, in a telephone call. If eligible, patients will be sent the *Patient Information Sheet*, re-contacted and, if interested in participating, invited to attend an appointment with the research nurse to complete the *Informed Consent*, *Demographics and Randomisation CRF*, and the *Baseline Measures CRF*. Informed consent will be obtained.

Demographic characteristics of age, type of IBD, childbirth, history of perianal fistula and any related surgery, other colorectal surgery, and current disease activity and medications, will be recorded prior to randomisation. The patient will then be entered into the randomisation database and told whether they are in group 1 (nurse + booklet) or group 2 (booklet alone). Hospital consultants will be informed of the patient’s participation in the study via an entry made in the patient’s notes; the patient’s general practitioner will be informed via a letter.

### Assignment for intervention

#### Allocation

Consenting participants will be randomised by computer-generated online block randomisation to either group 1 (IBD nurse CONSULT + BOOKLET) or group 2 (BOOKLET alone), stratified by centre and presence/absence of an ileo-anal pouch (which is associated with a high rate of FI), and using randomised blocks of two, four or six. Randomisation will be designed and managed by the PCTU.

#### Blinding

Participating centres will be blinded to sequence allocation until they log into the system and confirm participant eligibility. Blinding of participants and IBD nurses is not possible. The trial statistician will be blinded to treatment allocation during data analysis. We have not specified any permissible circumstances for unblinding in this low-risk trial.

### Data collection, management and analysis

#### Data collection

Data will be collected by a self-completed booklet of questionnaires. Study outcome measures will be recorded at baseline (booklet contains 19 printed pages) and at 6 months after randomisation (booklet contains 26 printed pages as it includes study-specific outcome tools and health economic data). Acceptability of these outcome booklets has been verified by our Patient and Public Involvement (PPI) Group. Preliminary piloting has found these take 20–30 minutes to complete and our four PPI representatives have commented that this is not unduly burdensome. Six months after randomisation, the outcome measures will be sent by post with two postal reminders for non-responders after 3 and 5 weeks.

#### Data management

Electronic study data will be pseudonymised, stored on a secure server and accessed via a secure network. Access is restricted to authorised personnel only and via secure, password-controlled, role-based access. All identifiable data stored in paper files will be managed securely in restricted-access, lockable containers at the individual participating sites. All authors will have full access to the final dataset. Following study closure, all data and study documentation will be archived at the sponsor site and held for 10 years, in accordance with sponsor requirements.

The PCTU standard operating procedures for data entry, quality control, data extraction and database freeze will be followed, and are available on request. All outcome measures are self-reported and will be returned to the recruitment site for inputting by the trial manager. The data management plan (available on request from the lead author), which includes source data verification and instructions for data storage, will be followed. After data cleaning, the database will be frozen and analysed by the study statistician, who will be blinded as to group allocation.

Outcome measures (see above) will be recorded at baseline and at 6 months following randomisation. There will be no additional follow-up. The study endpoint will be the date of database lock following data inputting, cleaning and checking. There will be no further quantitative data collection after this point.

#### Statistical methods

Primary and secondary outcome measures at baseline along with recruitment rates, session attendance, and withdrawal from the intervention will be presented as means and standard deviation for approximately normally distributed continuous variables, medians and interquartile ranges for non-normally distributed variables, and frequencies and percentages for categorical variables. Estimates of treatment effect on primary and secondary outcomes at the follow-up assessments will use an intention-to-treat (ITT) framework, implemented using a regression model, adjusting for baseline values of the outcome, centre, and presence of an ileo-anal pouch. ITT analysis will include all participants with outcome data regardless of adherence to the intervention. Where participants wish to withdraw from the intervention, we will attempt to retain them in the data collection, unless they express a wish to be withdrawn completely.

Also reported:

1. Comparison between the groups on secondary outcome measures, although the study is not powered to detect significant differences.
2. Sub-group analysis, including any apparent difference in response according to type of IBD (Crohn’s vs. other forms of IBD), childbirth (any vaginal delivery vs. none)
3. The impact of experiencing disease flare-up (defined as needing to escalate or initiate medication doses) on benefits of intervention
4. Health economic analysis (below)
5. Process data from study-specific questionnaire and IBD nurse records on which interventions were used
6. Qualitative data (see below)

#### Health economic analysis

The health economics evaluation will estimate the costs associated with the delivery of the nurse-led FI management programme to patients with IBD, and compare the use of health care resources by patients receiving the two FI management interventions: (i) booklet plus nurse consultations and (ii) booklet alone. The analysis will be conducted from the perspective of the NHS and Personal Social Services. Economic evaluation methods will adhere, as far as possible, to the NICE Guide to the Methods of Technology Appraisal 2013 [30].

The cost of delivery of the nurse-led FI management programme will include: the cost of booklet development and production; the cost of training IBD nurses; the cost of consultations with IBD nurses. In addition to the cost of delivery of the FI management programme, the costs of health care services use by participants will be studied. These costs will cover primary care services including consultations, medication, investigations and referrals to secondary care. Secondary care services will include outpatient appointments, inpatient episodes and accident and emergency department (A&E) visits. A resource-use questionnaire asking participants about their use of health care services and medication has been developed using the online Database of Instruments for Resource Use Measurement [31]. Primary and secondary care unit costs will be derived from the UK Unit Costs of Health and Social Care [32]. The unit costs for secondary care will be based on the UK National Schedule of Reference Costs [33]. The unit costs for medication will be obtained from the Prescription Cost Analysis database [34]. Costs associated with the two FI management strategies will be presented in a disaggregated format with effectiveness outcomes (cost-consequences analysis). The estimated mean costs for each group will be presented alongside the mean estimates of primary and secondary outcomes.

### Monitoring

#### Study management and access to data

The trial sponsor is London North West Hospitals NHS Trust, UK (contact Dr Alan Warnes: alan.warnes@nhs.net). The sponsor will provide insurance and access to compensation for participants who suffer harm.

The Trial Oversight Committee deliberates the practical and logistical aspects of the study. The committee is comprised of all co-applicants (co-authors of this manuscript). This committee will have access to the final dataset with no restrictions.

The Trial Management Group oversees the study to ensure fidelity to PCTU, sponsor, Research Ethics Committee and protocol requirements. The group is comprised of the chief investigator (CI), the trial manager, statistician, database manager and quality monitor, and a representative of the sponsor.

The Trial Steering Committee provides independent monitoring of the study, and is comprised of an independent chair, statistician, clinician, trial manager and patient representative. This committee also substitutes for a Data Monitoring Committee in this low-risk study.

The Patient and Public Involvement Group, supported by the trial manager, will contribute throughout the study to the design of patient-facing materials, and to analysis and report writing. It is comprised of a chair and four members, all of whom are patients with IBD.

#### Harms

This is a very low-risk study as no drug, medical device or surgical intervention is being trialled. However, the CI may take urgent safety measures to ensure the safety and protection of the clinical trial subjects from any immediate hazard to their health and safety.

The responsible medical consultant will be notified of their patient’s participation in the study, and will also receive an instruction sheet which lists all expected adverse events, and instructions to contact the CI in the event of any unexpected, potentially related adverse events. Expected potential adverse events for this study are:

1. Flare-up of disease activity (score of 5 or more on the Harvey Bradshaw index or the simple clinical colitis activity index)
2. Some degree of emotional distress

All adverse events will be recorded in the Adverse Events Log. Unexpected adverse events will be reported to the sponsor and the Research Ethics Committee within 24 hours.

#### Auditing

A trial monitor employed by the PCTU and independent from the investigators and the sponsor will monitor sites 6-monthly and in any case where concerns are raised by any member of the team.

#### Nested qualitative study

Participants consenting to the trial might be invited for interview. They will have received information about the interviews in the trial *Patient Information Sheet*. Separate informed consent will be sought for the face-to-face, semi-structured interviews with a purposive sample of approximately 20 participants (or until apparent data saturation), ten from each arm of the RCT. The interview sub-sample will include both genders, a range of ages, both IBD diagnoses and those successful and not successful in improving FI, to better understand patient perspectives on the intervention.

All participating IBD nurses will also be interviewed to understand their experience of delivering the interventions and to inform future adaptation and delivery of the intervention in clinical practice. A purposive sample of ten clinicians (gastroenterologists and IBD nurses not delivering the intervention) will be interviewed to ascertain their views of the interventions and ease of incorporating interventions for FI into routine IBD care. Four IBD service managers will be interviewed for views on incorporating a continence service into IBD services. All staff will receive the *Participant Information Sheet – Staff Interviews*. Consent will be obtained.

### Qualitative analysis

Interviews will be digitally recorded, anonymised, and professionally transcribed verbatim. Original audio files and file transcripts will be stored on a secure server at King’s College London in a protected file accessible only by the trial manager. The transcriber will delete her copy of each audio file once transcription is complete. Data will be analysed using a pragmatic thematic analysis [35]. Two researchers will code transcripts independently and then compare and refine resulting codes and themes in discussion. The emergent themes will form the basis of analytical interpretation.

### Ethics

The CI has obtained approval from a recognised Research Ethics Committee (National Research Ethics Committee London-Hampstead, project reference 154290, approval reference number: 15/LO/0051). The study will be conducted in accordance with the recommendations for physicians involved in research on human subjects adopted by the 18th World Medical Assembly, Helsinki 1964 (including later revisions), the UK Research Governance Framework for Health and Social Care 2005 and subsequent amendments, Good Clinical Practice Guidelines 1996, and the UK Data Protection Act 1998. Important protocol amendments will be communicated to the Research Ethics Committee and study sponsor by the CI.

### Consent

Potential participants will receive a full explanation of the study, be given the patient information sheet and have at least 24 hours to consider whether to take part. Written consent will be secured from recruited participants by an appropriately trained research nurse or clinician, or clinical trials practitioner, using the *Patient Consent Form* (Additional file 1) during a baseline appointment prior to the patient entering the trial and being randomised. Separate informed consent will be taken for interviews (see Additional file 2).

### Confidentiality

The CI will preserve the confidentiality of participants taking part in the study and will work in accordance with the Data Protection Act 1998, NHS Code of Confidentiality and any relevant NHS Trust organisational policies and NHS Caldecott Principles.

### Post-trial care

When the trial is completed, patients dissatisfied with the outcome will be offered referral to specialist continence services for investigation and treatment (routine NHS care). Booklet group participants will be offered the opportunity to receive the IBD nurse + Booklet intervention after completion of the outcome questionnaires at 6 months post-randomisation, according to local capacity to deliver. Numbers taking up referral or post-RCT intervention will be monitored and outcome of this routine specialised clinical care cohort will be assessed.

### Dissemination

It is planned to publish the results of this study in a scientific journal. Participants will be offered a lay summary of results. The co-applicants will be authors of this manuscript. There are no restrictions on publication of results from the sponsor or funder.

## Discussion

As FI is a potentially reversible symptom, with good evidence for what works for FI in other people and strong national guidance, there is potential to make a major difference to patients and produce high-quality evidence which could be rolled out across the NHS at relatively modest cost. It is important to clinicians as currently they do not address the issue of FI in IBD and have the potential to extend their services to address aspects that bother patients in more holistic care than currently offered. Current clinical services for IBD focus almost entirely on treatment of active disease and gut inflammation, with (to date) very limited attention to other symptoms and problems which stop patients working, participating in society and having a good quality of life. Our qualitative work and data on which interventions patients engage in and why will inform the way services are configured in the future. With a growing number of people living with long-term IBD there are many who could benefit from this research.

Relatively few high-quality studies of conservative interventions in inflammatory bowel disease have been conducted. We have collaborated closely with patients to design this study to meet their needs. Blinding to this behavioural intervention is not possible, but we have selected self-report outcome measures with a degree of objectivity. There is genuine equipoise between the booklet only and booklet plus nurse arms, and the study will determine if additional support from a nurse is a crucial element in implementing advice.

### Trial registration

This trial is registered on the publicly accessible registry (clinitrials.gov.uk: NCT02355834).

## Trial status

This protocol (version 4.0, dated 8 April 2015) is that approved by the Research Ethics Committee on 15 April 2015. Recruitment to the RCT commenced on 11 May 2015 and should complete by the end of February 2017. The study report will be available at the end of 2017. Reporting of this protocol conforms with the Spirit checklist (www.spirit-statement.org/wp-content/uploads/2013/01/SPIRIT-Checklist-download-8Jan13.pdf, accessed 05.10.15) as required by Trials (see Additional file 3).

## Additional files

Additional file 1:
**Consent form for RCT.** (PDF 121 kb) Additional file 2:
**Consent form for interviews.** (PDF 116 kb) Additional file 3:
**Spirit checklist.** (DOC 121 kb)

## Abbreviations

A&E: Accident and emergency department
CI: Chief investigator
CRF: Case report form
FCP: Faecal calprotectin
FI: Faecal incontinence
IBD: Inflammatory bowel disease
ITT: Intention to treat
NHS: National Health Service
NICE: National Institute for Health and Clinical Excellence
PCTU: Pragmatic clinical trials unit
PPI: Public and patient involvement
QALYs: Quality-adjusted life years
RCT: Randomised controlled trial
