# Supplementary material for: Faecal incontinence intervention study (FINS): self-management booklet information with or without nurse support to improve continence in people with inflammatory bowel disease: study protocol for a randomized controlled trial
Source: Trials. 2015 Oct 6;16:444. doi: 10.1186/s13063-015-0962-0 (PMC4594995; doi:10.1186/s13063-015-0962-0)
Supplement: Additional file 1: — Consent form for RCT. (PDF 121 kb) [file 13063_2015_962_MOESM1_ESM.pdf]

Faecal Incontinence iNtervention Study

STUDY ID	

NHS SITE LOGO HERE

REC Number: Study site (*please circle*): STM GSTT BTL JRO

CONSENT FORM (RCT)

Study title: Improving continence in people with inflammatory bowel disease: active case-finding and a randomised controlled trial

Chief Investigator: Professor Christine Norton

Please initial box

1. I confirm that I have read and understood the ‘Patient Information Sheet – RCT and Interviews’ [Version 2.0, dated 8.04.2015] for the above study and have had the opportunity to ask questions. ☐

2. I understand that I cannot choose whether I am allocated to Group 1 (IBD nurse + self-help booklet) or Group 2 (self-help booklet alone). ☐

3. I understand that my identity will be known to the research nurse who screens me for this study, to the IBD Nurse if I am allocated to Group 1, and to the interviewer and the chief investigator if I participate in the Phase 3 interviews. ☐

4. I understand that my data will be anonymised before it is shared with any other member of the research team, or used in publications or presentations. ☐

5. I understand that my medical notes may be looked at by the IBD Consultant or nurse, the research nurse, the trial manager, or auditors from the Clinical Trials Unit. I give permission for these individuals to have access to my records where it is relevant to my taking part in this research study. ☐

6. I agree to provide a stool sample to test for faecal calprotectin if I have not had a blood test for CRP levels within the 3 months prior to today ☐

7. I understand that my participation is voluntary, that I am free to withdraw at any time, without giving any reason, and that my medical care or legal rights will not be affected. ☐

8. I understand that if I do withdraw, or lose mental capacity to continue taking part and am withdrawn by the study team, any data already collected from me before withdrawal will be retained and used by the research team for this study unless I state otherwise. ☐

9. I agree to any members of this research team using my anonymised date again in future studies, without the need to seek further permission from me. ☐

10. I agree to my GP being informed that I am taking part in this study ☐

11. I agree to take part in the RCT phase of this study. ☐

White copy to patient; Blue copy in patient’s notes; Pink copy in research site file

Name of Patient	Date	Signature
Research nurse	Date	Signature
Name of person taking consent (if not research nurse)	Date	Signature

White copy to patient; Blue copy in patient’s notes; Pink copy in research site file
