# Supplementary material for: Faecal incontinence intervention study (FINS): self-management booklet information with or without nurse support to improve continence in people with inflammatory bowel disease: study protocol for a randomized controlled trial
Source: Trials. 2015 Oct 6;16:444. doi: 10.1186/s13063-015-0962-0 (PMC4594995; doi:10.1186/s13063-015-0962-0)
Supplement: Additional file 2: — Consent form for interviews. (PDF 116 kb) [file 13063_2015_962_MOESM2_ESM.pdf]

Faecal Incontinence iNtervention Study

STUDY ID	

NHS SITE LOGO HERE

REC number: 15/LO/0051

CONSENT FORM (INTERVIEWS)

Study title: Improving continence in people with inflammatory bowel disease: active case-finding and a randomised controlled trial

Chief Investigator: Professor Christine Norton

Please initial box

1. I confirm that I have read and understand the Patient Information Sheet – RCT and Interviews (Version 2.0, dated 8.04.2015) for the above study and have had the opportunity to ask questions. ☐
2. I agree to my interview being audio-recorded, and understand that this recording will be transcribed by a third party (professional) transcriber. ☐
3. I understand that my identity will not be known to anyone other than the person who interviews me, and to Professor Norton, and my details will be treated confidentially. ☐
4. I understand that before my interview data is shared with any other member of the research team, or incorporated in any publication, anything which might identify me will be removed. ☐
5. I understand that my medical notes may be looked at by the trial manager / interviewer, and individuals from regulatory authorities where it is relevant to my taking part in this research. I give permission for these individuals to have access to my records. ☐
6. I understand that my participation is voluntary and that I am free to withdraw at any time, without giving any reason, without my medical care or legal rights being affected. ☐
7. I understand that if I withdraw from the study, or lose mental capacity to continue taking part am withdrawn by the study team, any data already collected from me before withdrawal will be retained and used by the research team for this study unless I state otherwise. ☐
8. I agree to any members of this research team using my anonymised data again in future studies, without the need to seek further permission from me. ☐
9. I agree to take part in the interview phase of the study. ☐

Name of Patient	Date	Signature
Name of Person taking consent (if different from researcher)	Date	Signature
Researcher	Date	Signature

White copy to patient; Blue copy in patient's notes; Pink copy in research site file
